# A case of germline mosaicism for a 7q32.1q33 deletion in a sperm donor: consequences on pregnancy follow-up and recommendations

**DOI:** 10.1186/s12610-020-00113-5

**Published:** 2020-10-02

**Authors:** Celine Chalas, Aline Receveur, Nelly Frydman, Nathalie Massin, Gerard Tachdjian, Veronique Drouineaud, Alexandra Benachi, Catherine Patrat, Francois Michael Petit

**Affiliations:** 1 Laboratoire d’Histologie-Embryologie-Biologie de la Reproduction - CECOS, Hôpital Cochin, AP-HP, Centre Université de Paris, F-75014 Paris, France; 2 Laboratoire de cytogénomique, Hôpital Antoine Béclère, AP-HP, Université Paris Saclay, cedex, F-92141 Clamart, France; 3 Laboratoire d’Histologie-Embryologie-Cytogenetique- CECOS, Hôpital Antoine Béclère, AP-HP, Université Paris Saclay, cedex, F-92141 Clamart, France; 4 Faculté de médecine de Bicêtre, Université Paris-Saclay, F-94270 Le Kremlin Bicêtre, France; 5 Service de gynécologie et obstétrique, Centre Hospitalier Intercommunal, F-94010 Créteil, France; 6 Service de gynécologie et obstétrique, Hôpital Antoine Béclère, AP-HP, Université Paris Saclay, cedex, F-92141 Clamart, France; 7 Université de Paris, U 1016, Institut Cochin, F-75014 Paris, France; 8 Laboratoire de génétique moléculaire, Hôpital Antoine Béclère, AP-HP, Université Paris Saclay, cedex, F-92141 Clamart, France

**Keywords:** Germline mosaicism, Chromosomal rearrangement, FISH, Spermatozoa, Sperm donation, Mosaïque germinale, Réarrangement chromosomique, Spermatozoïde, Don de spermatozoïdes

## Abstract

**Background:**

Germline mosaicism is considered to be a rare event. However, its occurrence is underestimated due to the limited availability of germ cells. The genomic variations that underlie this phenomenon comprise single nucleotide polymorphism (SNPs), copy number variations (CNVs) and aneuploidies. In the case of CNVs, deletions are more frequent in the paternal germline while duplications are more commonly maternal in origin. Germline mosaicism increases with paternal age as the risk of SNPs increase with the number of germ cell divisions. We here report a case of germline mosaicism in the spermatozoa of a donor that resulted in one pathological pregnancy.

**Results:**

Straws from the same sperm donor were provided to seven recipient couples, resulting in four pregnancies. Second trimester ultrasound analysis revealed bilateral talipes equinovarus associated with growth retardation in one of these pregnancies. Array-comparative genomic hybridization (CGH) carried out after amniocentesis revealed a 4 Mb deletion in the 7q32.1q33 region. The blood karyotypes and array-CGHs were normal in the mother, as well as in the donor. However, the microsatellite profile indicated a paternal origin. Fluorescent in situ hybridization (FISH) analysis of the donor’s spermatozoa revealed the same chromosomal rearrangements in 12% of the spermatozoa population. Due to the documented risk of mental retardation associated with genomic rearrangements in the same region, the couple decided to terminate the pregnancy. Amniocentesis was performed in the other couples, which yielded normal FISH analysis results.

**Conclusions:**

Several cases of germline mosaicism have been reported to date, but their frequency is probably underestimated. Moreover, it is important to note that germline mosaicism cannot be ruled out by conventional cytogenetic screening of blood cells. This case highlights the need for close follow-up of every pregnancy obtained through gamete donation, given that the occurrence of germline mosaicism may have major consequences when multiple pregnancies are obtained concomitantly.

## Résumé

**Contexte:**

La mise en évidence d’une mosaïque germinale est. un événement rare mais probablement sous-estimé du fait de l’accès limité aux cellules germinales. Les variations génomiques caractéristiques de ce phénomène peuvent être des single nucleotide polymorphismes (SNPs), des copy number variations (CNVs) ou des aneuploïdies. Dans le cas des CNVs, les délétions sont plus fréquentes dans la lignée germinale paternelle tandis que les duplications sont plus fréquemment d’origine maternelle. Le risque de mosaïcisme germinal augmente avec l’âge paternel de part une augmentation du risque de SNPs associée à la division constante des cellules germinales pendant toute la vie d’un homme. Nous rapportons ici un cas de mosaïque germinale chez un donneur de spermatozoïdes ayant entraîné la survenue d’une grossesse pathologique.

**Résultats:**

Les paillettes d’un même donneur de spermatozoïdes ont été attribuées à sept couples receveurs permettant l’obtention de quatre grossesses évolutives. Pour l’une d’entre elle, l’échographie du deuxième trimestre a permis d’identifier chez le fœtus des pieds bots associés à un retard de croissance intra utérin. L’analyse par hybridation génomique comparative (CGH)-array après amniocentèse a révélé une délétion de 4 MB dans la région 7q32.1q33. Les caryotypes sanguins et les analyses par CGH-array étaient normaux chez la mère et le donneur. Cependant les profils de microsatellites ont montré une origine paternelle du chromosome délété. Une analyse par fluorescent in situ hybridization (FISH) des spermatozoïdes du donneur a révélé la présence de la même délétion dans 12% des spermatozoïdes étudiés. Etant donné le risque de retard mental associé à des remaniements chromosomiques dans cette même région, le couple a préféré interrompre la grossesse. Une amniocentèse a été réalisée pour les autres grossesse en cours et n’a retrouvé aucune anomalie.

**Conclusions:**

Plusieurs cas de mosaïques germinales ont été rapportés mais leur fréquence réelle reste probablement sous-estimée. En effet, un mosaïcisme germinal ne peut être détecté par les techniques de cytogénétique conventionnelle sur sang. Ce cas illustre la nécessité d’un suivi en temps réel des grossesses obtenues par don de spermatozoïdes étant donné que la survenue d’une grossesse pathologique peut avoir un retentissement sur les autres grossesses issues du même donneur.

## Background

Mutations generate sequence diversity and they provide a substrate for selection. However, some of them can be deleterious and give rise to a range of diseases. Classically, genomic variations are generally considered to be de novo if they are not found in the asymptomatic parent’s blood cells. Moreover, even when both parents have negative genetic test results, recurrence of the same genomic variation in siblings is still possible. The latter can be explained either by genetic hotspots or by germline mosaicism.

The genomic variations characteristic of this phenomenon can be single nucleotide polymorphisms (SNPs), copy number variations (CNVs) or aneuploidies. Aneuploidies are the most frequent chromosomal abnormalities in the first stages of embryonic development and they can affect any chromosome [1]. SNPs are more frequent in male germ cells than in female germ cells and they increase with paternal age [2]. Indeed, the spermatogonial stem cell pool is maintained through a high rate of cell division, with hence a higher risk of errors from mitosis as the individual ages, while the oogonial stem cell pool is established before birth. CNVs can occur through different mechanisms such as DNA repair or homologous recombination. A recent study has indicated that deletions frequently have a paternal origin, while duplications often have a maternal origin [3]. Regardless of the genomic variation, it is nearly impossible to confirm maternal mosaicism in germ cells due to the limited amount of material available. To date, cases of maternal germline mosaicism have been reported using indirect microsatellite analysis of blood cells [4]. By contrast, it is easier to examine paternal germline mosaicism on spermatozoa. Next-generation sequencing or micro-droplet digital polymerase chain reaction (PCR) can be used to detect SNPs [5, 6], whereas fluorescent in situ hybridization (FISH) on spermatozoa is appropriate for CNV detection [7].

Following one abnormal pregnancy from in-vitro fertilization (IVF) with a sperm donor, we identified a 4 Mb deleterious deletion of the 7q32.1q33 region in the fetus by array-comparative genomic hybridization (CGH) and FISH. Using FISH with the same probe on the spermatozoa of the donor, we confirmed the presence of a germline mosaicism in his spermatozoa. This finding highlights the importance of germline mosaicism identification following an abnormal pregnancy in the context of sperm donation, particularly since multiple pregnancies can be obtained concomitantly.

## Material and methods

### Sperm donation

Sperm donation is free and anonymous according to French law. Sperm donor must be aged less than 45 years old. A blood test for HIV1&2 (human immunodeficiency virus), HTLV1&2 (human T cell leukemia/lymphoma Virus), HBV (hepatitis B virus), HCV (hepatitis C virus), CMV (cytomegalovirus) and syphilis is carried out at the time of the sperm donation and 6 month later. Blood karyotyping is performed and a family health survey is conducted to identify heritable diseases. A pool of semen straws is generated and can be provided to several couples according to phenotypic criteria once the donation has been biologically approved. In accordance with French law, up to ten children can be born from the same donor.

### Cytogenetic analysis

Conventional cytogenetic analysis was carried out either on cultured amniotic fluid cells or on blood cells, using RHG and GTG banding according to standard protocol.

Array-CGH was performed on DNA extracted from uncultured amniotic fluid cells, using a 2x105K oligo platform (Agilent Technologies, Les Ulis, France) according to the manufacturer’s protocol.

FISH was performed using a Bacterial Artificial Chromosome (BAC) locus-specific probe (RP11-192 N3) and a sub-telomeric control probe (sub-telomere 7p) hybridized overnight with the ThermoBrite® system (Leica Microsystemes SA, Nanterre, France). The slides were washed in 2X Saline-Sodium Citrate (SSC) solution/0.1% NP40 solution and mounted with 4′,6-diamidino-2-phenylindole (DAPI) solution. The risks of error in the exclusion of chromosomal mosaicism, were calculated with 0.95 confidence limit and according to Hook’s tables [8]. FISH analysis was performed on 50 interphase and 2 mitotic cells using maternal blood cells (risk error of 6%) and on 100 interphase and 25 mitotic cells using the donor’s blood cells (risk error of 3%).

### Microsatellite analysis

The parental origin of the chromosomal rearrangement was determined using microsatellite markers. Briefly, after DNA extraction from uncultured amniotic fluid cells or peripheral leukocytes, microsatellite markers were PCR-amplified using fluorescent primers and then separated on a 3130 ABIPRISM® genetic analyzer (Applied Biosystems, Courtaboeuf, France). Fragment lengths were determined using GeneMapper™ software (Applied Biosystems) by comparison with the GeneScan™ 500LIZ™DNA ladder (Applied Biosystems).

### FISH analysis of spermatozoa

Frozen spermatozoa were washed two times in phosphate buffered saline (PBS) solution and fixed in Carnoy’s solution. After being spotted on the slide, the spermatozoa heads were decondensed using 1 N NaOH solution for 1 min and 30 s. After control of a sufficient degree of sperm head decondensation using the microscope, the spermatozoa were sequentially dehydrated in alcohol (70% followed by 90% and finally, 100%). FISH on the spermatozoa was carried out using the same probes and conditions as outlined above. The spermatozoa were examined using an epifluorescent microscope according to strict criteria, including individual and well-delineated sperm heads, the color, the size and the intensity of spots as previously described [7]. The BAC and sub-telomere probes had previously been tested on spermatozoa from a fertile donor and no deletions were found (1000 sperm heads counted per probe). More than 1000 spermatozoa were scored and the significance threshold was set at 1% (according to Hook’s tables [8]).

## Results

### Sperm donor

A 43-years-old male, whose motivation for sperm donation was based on altruistic considerations, attended the center for study and preservation of eggs and sperm (CECOS) to donate his semen. His blood analysis results for infectious diseases were negative and his karyotype carried out on blood cells was normal (46XY). At the time of the donation, he was living with his partner and had a healthy five-years-old son. Moreover, his familial health survey did not reveal any personal or familial diseases.

### Recipient couples

Seven couples received sperm from the straws of this donor, leading to five pregnancies. However, one of these five pregnancies ended in an early miscarriage.

**Fig. 1:**
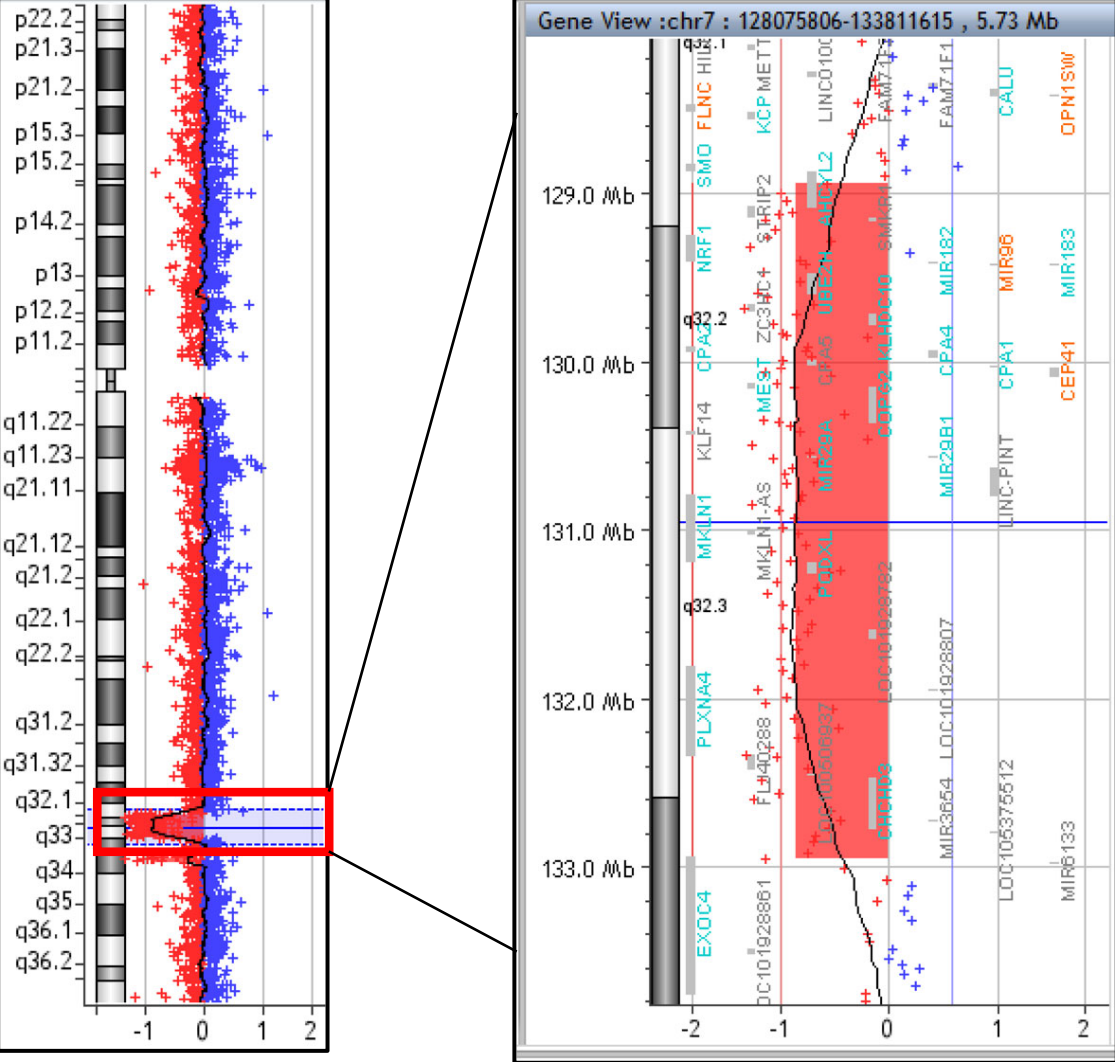
Identification of the interstitial deletion by array-comparative genomic hybridization (CGH) on amniotic fluid. Array-CGH was performed on DNA extracted from uncultured amniotic fluid cells identifying a 4 Mb interstitial deletion of the long arm of chromosome 7 in the 7q32.2q32.3 region. This deletion located in the 128936178–132951244 region encompasses 22 genes referenced in the Online Mendelian Inheritance in Man (OMIM) database (www.omim.org)

In the most advanced pregnancy (i.e., the one closest to term), the second trimester ultrasound exam revealed that the fetus harbored severe bilateral talipes equinovarus associated with intrauterine growth retardation. Subsequently, an amniocentesis was performed at 24 weeks + 6 days of amenorrhea. Spinal muscular atrophy and myotonic dystrophy 1 were ruled out by molecular analysis. Standard karyotyping did not detect any visible chromosomal abnormalities. However array-CGH revealed a 4 Mb deletion on the long arm of chromosome 7 (arr[GRCh37] 7q32.1q33(128936178_132951244)× 1, Fig. 1), which was confirmed by FISH on cultured amniotic fluid cells. Due to the documented association of chromosomal abnormalities involving this deletion and fetal malformations, the couple decided to opt for pregnancy termination at 36 weeks + 5 days of amenorrhea in accordance with French law.

### Pathological pregnancy investigation

A familial study using FISH analysis with the same probes was performed on the maternal and the donor blood cells. No anomalies were detected, thus suggesting either a de novo mechanism or a germline mosaicism.

**Fig. 2:**
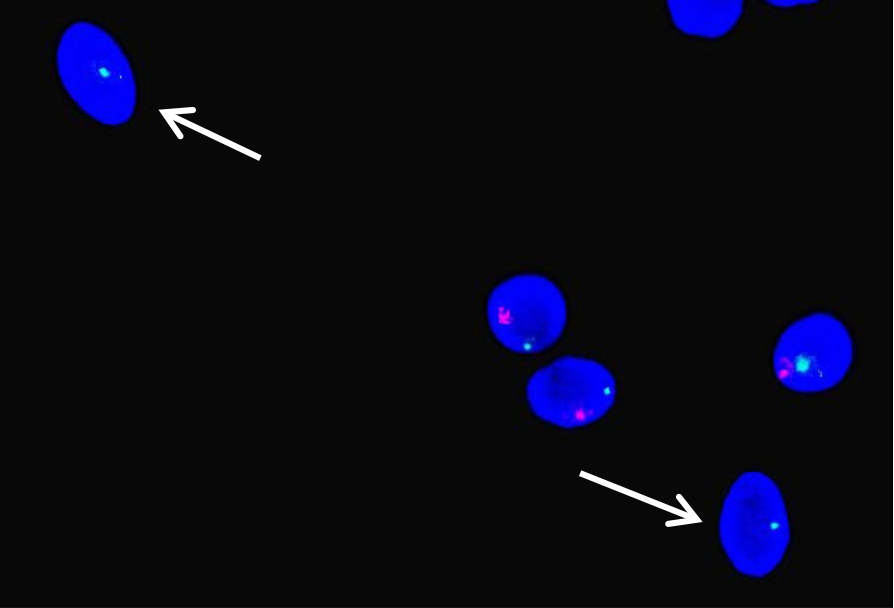
Identification of the germline mosaicism by fluorescent in situ hybridization analysis of the spermatozoa of the sperm donor. After sperm head decondensation, a locus specific probe at locus 7q32.3 (in red) and chromosome 7 control probe (in green) were co-hybridized. Sperm DNA was counterstained with DAPI (4′,6-diamidino-2-phenylindole). The arrows indicate the sperm heads carrying the deletion (one spot for the control probe and no spots for the specific probe)

To further assess the pathological mechanism involved, we probed for a paternal origin of the deletion based on microsatellite profiles. Three semen straws from the same sample were thawed for FISH analysis. The results of this analysis revealed that 132/1099 spermatozoa (12%) carried the deletion (Fig. 2).

Notably, none of the three other ongoing pregnancies were associated with a fetal anomaly based on ultrasound examination. However, this finding was not considered to be sufficient to exclude the presence of the deletion. After genetic counseling, two out of the three couples agreed to undergo amniocentesis. The consequent FISH analysis did not detect the 7q32.1q33 deletion. Finally, four healthy babies (including twins) were born from this donation.

The sperm donor was informed of the germline mosaicism found in his gametes.However, he declined genetic counseling because he no longer wished to have more children.

## Discussion

We here report a case of male germ cell mosaicism confirmed by FISH in spermatozoa. It involved a 4 Mb deletion on chromosome 7 (7q32.1q33) that was observed in 12% of the spermatozoa of a sperm donor who had previously fathered a healthy child. Unfortunately, no other tissues were available for analysis, and FISH analysis of sperm straws from different retrievals was not available to evaluate the variability of the germline mosaicism during the spermatogenic cycle.

Several genes implicated in the development and function of specific brain areas, including areas responsible for language processing and intellectual development, have been identified on the long arm of chromosome 7 [9–14]. There have been only few reports to date of deletions of the distal region of chromosome 7 and they involved isolated cases of de novo mutation in the region spanning 7q32 and 7q33–36 [9–14]. The 7q32 locus is known to be an autism susceptibility locus, containing both imprinted and non-imprinted genes, such as *UBE2H*, *CPA4/5, MEST, COPG2, KLF14, MKLN1* and *PODXL*. The *MEST/PEG1* maternally imprinted gene (mesoderm specific transcript/paternally expressed gene1) has been proposed to be a candidate gene for Silver Russel syndrome [15, 16]. These reports reassured the couple in their choice for a pregnancy termination.

Male germline mosaicism is probably underestimated in cases of mutation or CNV when only one descendant is involved. Recent studies have focused on the presence of SNPs in the male germline and their increased occurrence with paternal age [17]. Indeed, male germ cells undergo continuous genome replication throughout a man’s lifespan [18]. Rahbari et al have proposed that mutation can occur early during embryonic development, prior to the separation between the germ cells and the soma, resulting in mosaicism within both tissues. In this case, mosaicism can also be identified in the soma of the parent but at a low level, thereby explaining why the parent can be asymptomatic, or has milder symptoms. However, in some cases, no mosaicism can be detected in the blood cells or other tissues, thus suggesting that the mutation occurred after the separation of the soma and the germ cells, in post-primordial germ cells [18]. Yoon et al have modeled a mutation algorithm to explain the occurrence of mutations restricted to a cluster originating from the division of Ap spermatogonia. In this model, a punctual mutation of one Ap spermatogonia can be selected and amplified during cell proliferation, thereby giving rise to a cluster of mutated germ cells [19]. The mutation provides a selective advantage for the spermatogonial stem cells, favoring expansion of the cluster, also known as the selfish mechanism. This scenario is reported as paternal age effect and is associated with several developmental disorders such as Noonan and Costello syndrome, Apert syndrome and Achondroplasia [20].

Other cases of germline mosaicism in sperm donor have been described. Both Callum et al and Ejerskov et al have reported a sperm donor carrying a germline mosaicism for a deletion in the *NF1* gene, resulting in children afflicted with neurofibromatosis type 1 [21, 22]. Moreover, the report published by Ejerskov et al involved straws distributed internationally by a Nordic cryobank. Surprisingly, the bank was not able to determine the number of children born from this donor and had no information regarding their distribution in foreign countries.

## Conclusions

In conclusion, this case has prompted us to implement the following guidelines for sperm donations:

i. It is important to undertake an exhaustive genetic counseling with the donor to detect potential personal or familial genetic pathologies. This consultation is not foolproof, especially since donation is currently open to people without children. Moreover, germline mosaicisms, as it is the case for other illness, are not predictable and the recipients should be made aware that it is not possible to eliminate all risk. Thus, they cannot be fully shielded from the possibility of an accidental pathology.
ii. Any abnormal pregnancy or pathology in a fetus or a child born from a sperm donation must lead to an immediate suspension of straw distributions.
iii. Follow-up of pregnancies obtained by a sperm donation is an absolute requirement. As the donor gametes may be distributed to several couples at the same time, simultaneous pregnancies can be obtained. Since the CECOS ensures a comprehensive follow-up of the distributed straws, we were able to rapidly identify couples who received the same sperm samples and thus offer them prenatal testing for the identified mutation.
iv. Moreover, as soon as a chromosomal aberration is identified in a fetus but is not found in the parent’s blood cells, we would strongly recommend evaluation of its parental origin by microsatellite analysis. If paternal inheritance is found to be the case, germline mosaicism must be examined in the sperm; whereas in case of maternal inheritance, germline analysis is more challenging.

## Data Availability

The datasets used and/or analyzed during the current study are available from the corresponding author on reasonable request.

## Abbreviations

BAC: Bacterial Artificial Chromosome
CECOS: Centre d’Etude et de Conservation des Oeufs et du Sperme (Center for study and preservation of eggs and sperm)
CGH: Comparative genomic hybridization
CMV: Cytomegalovirus
CNV: Copy number variation
DAPI: 4′,6-diamidino-2-phenylindole
FISH: Fluorescent in situ hybridization
HBV: Hepatitis B virus
HCV: Hepatitis C virus
HIV: Human immunodeficiency virus
HTLV: Human T cell leukemia/lymphoma virus
IVF: In vitro fertilization
MEST/PEG1: Mesoderm specific transcript/paternally expressed gene 1
OMIM: Online Mendelian Inheritance in Man
PBS: Phosphate buffered saline
PCR: Polymerase chain reaction
SNP: Single nucleotide polymorphism
SSC: Saline sodium citrate
